# Comparative Study Highlights the Potential of Spectral Deconvolution for Fucoxanthin Screening in Live *Phaeodactylum tricornutum* Cultures

**DOI:** 10.3390/md20010019

**Published:** 2021-12-23

**Authors:** Sean Macdonald Miller, Raffaela M. Abbriano, Anna Segecova, Andrei Herdean, Peter J. Ralph, Mathieu Pernice

**Affiliations:** 1 Climate Change Cluster (C3), Faculty of Science, University of Technology Sydney, Sydney, NSW 2007, Australia; raffaela.abbrianoburke@uts.edu.au (R.M.A.); segecova.a@czechglobe.cz (A.S.); andrei.herdean@uts.edu.au (A.H.); peter.ralph@uts.edu.au (P.J.R.); mathieu.pernice@uts.edu.au (M.P.); 2 Global Change Research Centre, Academy of Science of the Czech Republic, 26101 Drasov, Czech Republic

**Keywords:** fucoxanthin, microalgae, green consumption, food consumption

## Abstract

Microalgal biotechnology shows considerable promise as a sustainable contributor to a broad range of industrial avenues. The field is however limited by processing methods that have commonly hindered the progress of high throughput screening, and consequently development of improved microalgal strains. We tested various microplate reader and flow cytometer methods for monitoring the commercially relevant pigment fucoxanthin in the marine diatom *Phaeodactylum tricornutum.* Based on accuracy and flexibility, we chose one described previously to adapt to live culture samples using a microplate reader and achieved a high correlation to HPLC (R^2^ = 0.849), effectively removing the need for solvent extraction. This was achieved by using new absorbance spectra inputs, reducing the detectable pigment library and changing pathlength values for the spectral deconvolution method in microplate reader format. Adaptation to 384-well microplates and removal of the need to equalize cultures by density further increased the screening rate. This work is of primary interest to projects requiring detection of biological pigments, and could theoretically be extended to other organisms and pigments of interest, improving the viability of microalgae biotechnology as a contributor to sustainable industry.

## 1. Introduction

Fucoxanthin is the most abundant marine carotenoid pigment, accounting for more than 10% of the total carotenoids produced naturally [1]. Fucoxanthin is of commercial interest for its antioxidant, anti-inflammatory, antibacterial, and anti-obesity characteristics [2,3,4,5], as well as having potential in inhibiting cancer cell growth [6]. Due to these beneficial properties, fucoxanthin is an established nutraceutical currently sourced from seaweed [7,8]. However, the marine diatom *Phaeodactylum tricornutum* (Bacillariophyta) exhibits up to 100 times higher fucoxanthin content (mg g^−1^ DW) than seaweed [5,9], which makes it an attractive alternative for commercial purposes. *P. tricornutum* has also been used extensively across a wide area of research and has full genome data [10]. This species has demonstrated genetic manipulability [11,12,13], which further supports its utility as a biofactory template for fucoxanthin. Fucoxanthin in *P. tricornutum* can reach up to 59.2 mg g^−1^ under optimal growth conditions [14], and it has been improved by 69.3% (mg g^−1^ DW) in chemically-induced mutants [15] and by 45% per cell by introduction of the PSY (phytoene synthase) gene [16] compared to the wild-type strain.

Strategies that generate large mutant or transformant libraries (hundreds to thousands of clones) necessitate high-throughput methods to screen novel strains for pigments because the current benchmark of pigment detection is high-performance liquid chromatography (HPLC), which, while being accurate, is very expensive and time-consuming. Several approaches to screening fucoxanthin in *P. tricornutum* were investigated here and compared in terms of the accuracy of their prediction as compared to HPLC, as well as the effort invested to produce that result. Because fucoxanthin and chlorophyll *a* are associated with light harvesting, chlorophyll screening methods were included as fucoxanthin proxies alongside direct fucoxanthin quantifiers, and their assessment included both microplate reader and flow cytometer formats. Flow cytometry provides a means of analyzing samples at the resolution of individual cells, which negates effects from culture density and can be attached to fluorescence-activated cell sorting (FACS), while the microplate reader format provides an inexpensive and non-invasive way of analyzing many samples in a short period of time, therefore, both systems were included.

Three microplate reader methods and three flow cytometry methods were chosen for this study. First, two estimates for chlorophyll and fucoxanthin autofluorescence were measured using flow cytometry to investigate whether various excitation/emission gates could accurately predict average fucoxanthin content through analysis of single cells. A third flow cytometry method using Nile Red dye was included to investigate if dye fluorescence can improve the detection of fucoxanthin based on its relationship to lipids [17] and correlation to total carotenoids [15]. One approach for estimating chlorophyll in ethanol was tested as a proxy for measuring fucoxanthin in microplate format from Ritchie [18]. The second microplate reader method investigated in this study is theoretically similar to the equations from Ritchie [18], except that it aims to quantify fucoxanthin directly by using equations based on specific wavelength absorbance of pigment extracts related to fucoxanthin, rather than using chlorophyll as a proxy, found in Wang et al. [19]. The final microplate reader method is from Thrane et al. [20], developed from earlier work [21,22], and uses spectral deconvolution of absorbance spectra obtained from samples extracted using an organic solvent like ethanol. All samples were compared during exponential growth phase, as this is the easiest point to detect pigment content differences without compounding effects from media changes and cell senescence, and enables the screening methods with the best resolution to be highlighted accordingly.

This study aims to reconcile accuracy with practical considerations of screening methods, and to further optimize a promising method for high-throughput screening of fucoxanthin in *P. tricornutum*. Six published methods using two common pieces of analytical equipment were used to assess their correlation to HPLC, one being chosen for further optimisation based on this correlation as well as practical considerations like ease of use compared to cost and time investment. This work contributes to the field of microalgal biotechnology by improving the rate at which novel strains of *P. tricornutum* can be selected based on improved fucoxanthin content.

## 2. Results and Discussion

### 2.1. Culture Characteristics

**Figure 1:**
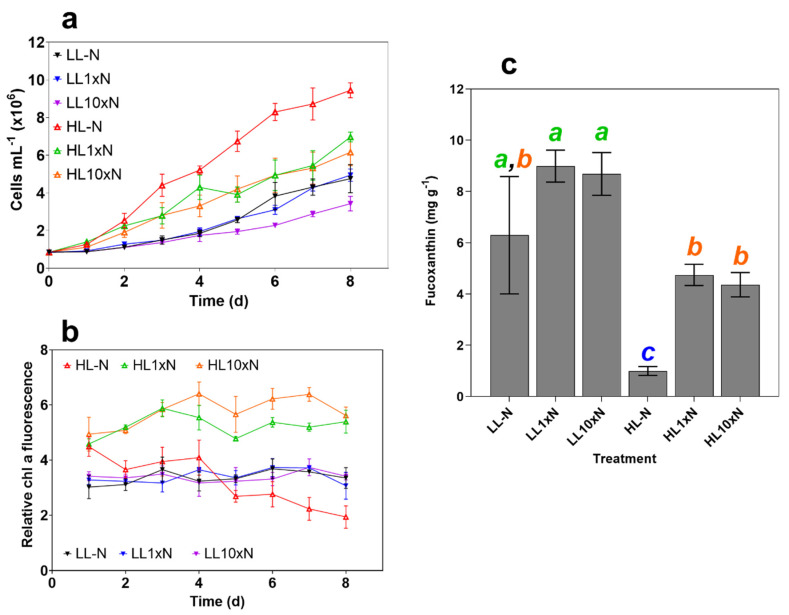
Measured culture characteristics over the 8-day experimental period. (**a**) Cell density in millions mL^−1^; (**b**) Relative chlorophyll *a* fluorescence (chlorophyll *a* fluorescence/culture absorbance at 750 nm), which was used to determine optimal experiment termination time point; and (**c**) fucoxanthin content of freeze-dried samples measured using HPLC (mg g^−1^). Treatment abbreviations are as follows: nitrate-free ASW media (-N), standard (1 × N) nitrate media or media with 10× nitrate (10 × N), and either 10 (LL) or 200 (HL) µmol photons m^−2^ s^−1^ of white light. Statistical significance was calculated using one-way ANOVA (*p* < 0.05) with letters denoting non-significant groupings. Error bars denote standard deviation (*n* = 3).

The high light without nitrate (HL-N) treatment cell growth was statistically significantly higher (*p* = 0.0005) than both other HL treatments (~9.5 × 10^6^ compared to an average 6.5 × 10^6^ cells mL^−1^) on the final day (Figure 1a). While the final pellet weights for all HL treatments were similar, the mean singlet forward scatter (FSC-A) of HL-N cultures was 50% less than the forward scatter of nitrogen-present HL treatments (*p* < 0.0001). Decreased cell size and volume due to nitrogen limitation has been observed previously [23] and explains the much higher cell density in HL-N treatments. Importantly, this indicates that *P. tricornutum* has impressive light stress tolerance mechanisms despite low or no nutrient availability, considering the analogous biomass productivity of HL treatments, which averages significantly higher (*p* = 0.0206) at up to two times that of the lowest LL biomass productivities.

Pigment content was measured by relative chlorophyll *a*, and the experiment was terminated on day 8 when relative chlorophyll *a* was significantly different (*p* < 0.0001, ~1.9 for HL-N compared to ~3.3 for all LL treatments and ~5.5 for HL treatments with nitrate supplementation, Figure 1b). LL cultures displayed very similar chlorophyll *a* autofluorescence over the experimental period, while there was a clear distinction between nitrogen replete and deplete conditions under HL.

As expected, fucoxanthin content was a function of irradiance, specifically, its role as a light-harvesting pigment was demonstrated by its negative correlation with photon availability. Nitrogen limitation decreased overall fucoxanthin content and productivity under 200 µmol photons m^−2^ s^−1^, while simply increasing the variability under 10 µmol photons m^−2^ s^−1^ (Figure 1c). These treatments provided a sufficient range of fucoxanthin contents to assess high-throughput screen viability.

### 2.2. High-Throughput Screen Analysis

Data for live culture chlorophyll *a* autofluorescence on microplate reader were not shown because there was no correlation to HPLC (R^2^ < 0.002). The microplate reader Nile Red fluorescence data displayed a negative correlation, which can be attributed to disturbance by media noise and other resolution issues, and these were therefore not considered reliable methods.

**Figure 2:**
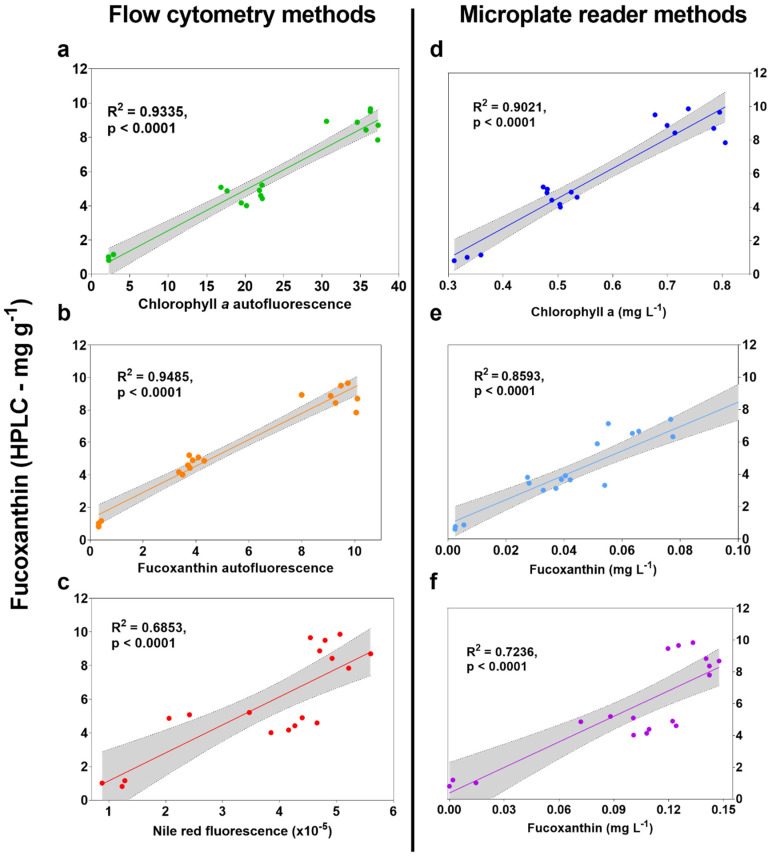
High-throughput screen results correlated to fucoxanthin (mg g^−1^) measured using HPLC. (**a**) Mean single cell chlorophyll *a* autofluorescence measured on flow cytometer using blue excitation wavelength of 488 nm with 690/50 nm optical filter, (**b**) mean single cell fucoxanthin autofluorescence measured on flow cytometer using yellow excitation laser at 561 nm with 710/50 optical filter, (**c**) mean single cell fluorescence measured on flow cytometer using blue excitation laser at 488 nm with 610/20 optical filter after dyeing with Nile Red, (**d**) chlorophyll *a* content (mg L^−1^) using equations for ethanol extracts from Ritchie (2008) on a microplate reader, (**e**) fucoxanthin content (mg L^−1^) for concentrated ethanol extracts from Wang et al. (2018) on a microplate reader, and (**f**) spectral deconvolution method from Thrane et al. (2015) using raw culture absorbance spectra on microplate reader. Units on x axes for d, e, and f are simply what the sources for each use to determine fucoxanthin content, while samples for all three were extracted herein using an equal weight of biomass, effectively making x-axis units the weight of fucoxanthin per unit weight of biomass (like HPLC).

Autofluorescence measurements using flow cytometry displayed the highest correlation to HPLC data with R^2^ = 0.9335 and R^2^ = 0.9485 for chlorophyll *a* (B690-50, Figure 2a) and fucoxanthin (Y710-50, Figure 2b), respectively. These were also higher than both Nile Red and the equations from Ritchie [18] and Wang et al. [19], which require either dyeing or extraction with an organic solvent. While chlorophyll *a* autofluorescence was included to estimate the B690-50 filter reliability at estimating fucoxanthin, using alternatives is likely to give more accurate predictions, as with the Y710-50 filter. Excitation at 561 nm with emission at 710 nm (Y710-50) has generally been avoided in the literature because there is little or no absorption of individual *P. tricornutum* pigments at these wavelengths. However, Premvhardan et al. [24] showed a shift in fucoxanthin absorbance when bound in Fucoxanthin-Chlorophyll Protein (FCP) to wider than 561 nm. Because the flow cytometry methods are measuring live cells rather than pigments in extracts, it is appropriate to assess fucoxanthin absorbance characteristics when bound to FCP. Also, an extra emission peak at 710 nm was observed by Fan et al. (2021) in *P. tricornutum*, who confirmed a relationship between emission intensity at 710 nm and fucoxanthin content measured using HPLC [25]. Using these wavelengths has the likely benefit of being able to measure fucoxanthin content by its relationship to cellular FCP rather than by chlorophyll content as a proxy, like when measuring extracts using equations from Ritchie et al. (2008) [18]. Despite the overall fluorescence intensity of Y710 being about 25% that of the B690 channel (data not shown), it appears sufficient as not only an accurate indicator, but more reliable in predicting fucoxanthin content than B690-50.

Measuring autofluorescence with flow cytometry is not only accurate for measuring fucoxanthin, it does not require extraction, dilution or dyeing, and this greatly improves screening time. Relevant to many projects that generate large mutant or transformant libraries, FACS can also be attached to this method to further improve screening and selection times, as also found in Gao et al. [17] and Fan et al. [25].

The use of Nile Red to correlate polar lipids with fucoxanthin has been performed previously using live cultures of *Tisochrysis lutea* with an R^2^ value of 0.88 [17], there is a correlation between Nile Red and total carotenoids in *P. tricornutum* [15]. A relationship to HPLC was found herein (R^2^ = 0.69) for *P. tricornutum* using the average single cell fluorescence of singlets after staining with Nile Red (Figure 2c). While a correlation was found, using different channels resulted in higher R^2^ values without the need to dye first.

Microplate reader formats displayed overall lower correlation to HPLC, with the maximum found using the method from Ritchie [18] (R^2^ = 0.9021, Figure 2d). 

In our initial analysis, the method from Wang et al. [19] displayed reduced correlation to HPLC at R^2^ = 0.0292 when the dilution of 1 × 10^6^ cells mL^−1^ was used. Verification cultures in the original source use a cell density between 20–100 × 10^6^ cells mL^−1^, which is likely to have contributed to the sensitivity and therefore accuracy reported therein (R^2^ ≥ 0.946). To improve the resolution of fucoxanthin predictions using the method from Wang et al. [19], experimental samples were analyzed again at higher cell concentration and an R^2^ value of 0.8593 was found (Figure 2e). Despite this method displaying a slightly lower correlation than in the original source (R^2^ = 0.8593 compared to R^2^ ≥ 0.946, see [19]), users performing equipment calibration can expect even better results and should keep in mind the changes to resolution when using low cell densities. 

Despite displaying a relatively high correlation of R^2^ = 0.7236 (Figure 2f), the spectral deconvolution method from Thrane et al. [20] did not require solvent extraction. This method also had the greatest potential for calibration and refinement, owing to its use of a much larger dataset and multiple programmable input scripts using RStudio version 1.3.1093 (RStudio, Boston, MA, USA). Therefore, the method was chosen for further optimisation. 

### 2.3. Optimisation of Spectral Deconvolution from Thrane et al. (2015)

The R scripts contained within Thrane et al. [20] were first tested using absorbance spectra obtained from uniformly-dilute ethanol-extracted experimental samples. It was noticed that predictions for fucoxanthin were either extremely low or zero. To allow the method to attribute lower wavelength spectral regions to the presence of fucoxanthin, pigments that are not present in *P. tricornutum*, or present in only minute amounts, were removed from the R script “gaussian.peak.parameters.txt” as well as from line 32 of the “pigments.function.R” script. It was found that removing all but the chlorophylls *a*, *c*1 and *c*2, and fucoxanthin from the method ensured detection of fucoxanthin in every sample. After removal of ‘unnecessary’ pigments and the reassigning of pathlength in the ‘Sediments.R’ file to z = 0.625 for microplate reader adaptation, the correlation between the script and the content of fucoxanthin detected using HPLC in mg g^−1^ was R^2^ = 0.801. 

**Figure 3:**
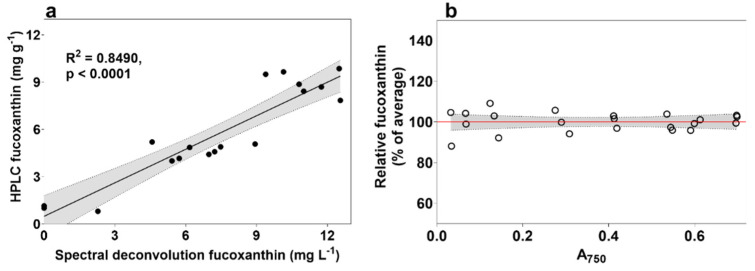
Correlation of modified spectral deconvolution method to fucoxanthin measured using HPLC. (**a**) Correlation after modifying fucoxanthin coefficients, and (**b)** results after normalizing absorbance spectra to culture density (absorbance at 750 nm) on a 384-well microplate.

The spectral qualities of a pigment are altered when measured in live cell thylakoid membranes compared to when measured as free-floating molecules in an organic solvent like ethanol. The cumulative effects of the silica frustule of *P. tricornutum*, the lipid layers associated with the thylakoid, effects from saltwater medium, and interference from other cellular components, are expected to have substantial effects on fucoxanthin spectral characteristics, so the ethanol-fucoxanthin coefficients used in the original source were replaced. Firstly, the *P. tricornutum* absorbance spectrum from cultures under LL+N (maximum fucoxanthin) were averaged and the spectrum between 400–550 nm was used to estimate new fucoxanthin Gaussian peaks using the “chl.b.fit.R” file from the original source. These new coefficients for peak height (“a”), wavelength (“xp”) and peak halfwidth (“s”) for live cultures were then used instead of the original coefficients for ethanol and used to compare the spectral deconvolution (mg L^−1^) to fucoxanthin as measured using HPLC (mg g^−1^), which resulted in an R^2^ value of 0.849 (Figure 3a).

The above method analysis and troubleshooting was performed with pre-diluted culture samples for ease of use and confidence of comparison. To explore the possibility of using microplate well cultures without equalizing by cell density, and to exclude potential effects from differences in individual cell volume amongst a population, an exponential-phase wild type *P. tricornutum* culture was diluted to various densities between approximately 0.5 and 20 × 10^6^ cells mL^−1^ (A_750_ between 0.03 and 0.7) and their predicted fucoxanthin values compared on a 384-well microplate using spectral deconvolution of individual wavelengths normalized by the absorbance at 750 nm (Figure 3b), using the following equation:

SpeDec Fx (mg L^−1^) = spectra [400 − 700 nm]/(Sample A_750_ − ASW A_750_)

The choice to move to 384-well microplates instead of 96-well microplates was made to further accelerate the screening rate. Fucoxanthin predictions across replicates were within 8% of each other when only including samples at optical density of 0.4 or above. The success of using the absorbance at 750 nm to normalize the results of the spectral deconvolution method relies on each wavelength of a culture spectrum (400–700 nm) being divided by the culture A750 after blanking to culture media absorbance at the same wavelength. This method allows multiple wells to be measured without prior dilution, removing the need to account for screen bias towards denser cultures, but does not account for increased fucoxanthin due to culture shading effects.

The benefit of using this method in comparison to flow cytometry is that the latter is expensive to purchase and run, requires skilled users, and most importantly cannot screen using microplate format without a high risk of cross-well contamination. The modified spectral deconvolution method from Thrane et al. [20] allows users to screen libraries of hundreds to thousands of individuals cost-effectively without removing microplate lids or culture volume to preserve sterility, which also enables temporal evaluation across growth curves and different growth conditions. Method benefits are outlined in Table 1.

## 3. Materials and Methods

### 3.1. Stock Culturing

Axenic *Phaeodactylum tricornutum* (CCAP 1055/1) stock cultures were obtained from the C3 culture collection and grown in Artificial Sea Water (ASW) medium (Darley & Volcani, 1969) under fluorescent light (200 µmol photons m^−2^ s^−1^) with a 24:0 light cycle in shaking Erlenmeyer flasks (95 rpm) kept at 21 °C. Treatment flasks were inoculated at 8.5 × 10^5^ cells mL^−1^ from cultures at exponential stage.

### 3.2. Experimental Design

Treatment flasks consisted of 250 mL conical flasks containing 100 mL of respective media. The highest fucoxanthin content was achieved with low light availability and high nitrate availability [14], so combinations of these two factors were used to maximise the expected culture fucoxanthin content range. Cells were inoculated into either nitrate-free ASW media (-N), standard (1 × N) nitrate media, or media with 10× nitrate (10 × N) from KNO3 and placed under either 10 (LL) or 200 (HL) µmol photons m^−2^ s^−1^ (Figure S1). To diminish variances in media composition introduced by pipetting culture volumes directly into experimental flasks, 30 mL culture aliquots were centrifuged and the pellets were then pipetted into experimental flasks (~2.5 × 10^7^ cells total). Three × 30 mL stock cultures were also centrifuged, washed once with ultrapure water, flash-frozen in LN2, lyophilised, and weighed to estimate a starting value for biomass in each experimental flask. The treatments were left until there was a detectable difference observed in the relative chlorophyll *a* fluorescence across treatments.

### 3.3. Sampling

Each day, 200 µL from each flask was transferred into a clear-bottom, black 96-well microplate (Corning Inc., Corning, New York, NY, USA), and a plate reader (Tecan Infinite M1000 Pro) was used to measure absorbance at 750 nm as well as chlorophyll *a* fluorescence using an excitation wavelength of 440 nm and emission wavelength of 680 nm. Cells were counted daily using the Cytoflex LX flow cytometer (Beckman Coulter, Brea, CA, USA) by separating singlets via gating within an XY plot of forward scatter (FSC) and side scatter (SSC). A second plot comparing forward scatter by cell area (FSC-A) to chlorophyll *a* fluorescence (excitation with blue laser at 488 nm with 690-50A optical filter) was constructed to separate singlets into live cells and dead cells/debris. 

At the conclusion of the experimental period, flask culture volumes were measured, centrifuged, and washed once with ultrapure water to reduce dissolved salts. They were then flash-frozen in liquid nitrogen and lyophilized before being kept at −80 °C for measurement using HPLC. The remaining flask media was kept at −30 °C for nitrate analysis.

Culture aliquots were diluted to 1 × 10^6^ cells mL^−1^, and pigment extraction of both diluted and undiluted culture aliquots was performed by centrifugation and replacement of supernatant with equal volume of absolute ethanol kept at −30 °C. Extraction was furthered using 3 × 3-s pulses of an ultrasonic homogenizer (Qsonica Q125) at maximum amplitude with centrifugation in between pulses to confirm blanching of pellets and thus complete extraction of cellular pigments. Extracts and raw culture, both diluted and undiluted, were used to attain data for the various screening methods by pipetting 200 µL into clear-bottom, black 96-well microplates and measuring on microplate reader and by flow cytometry.

### 3.4. Chlorophyll and Fucoxanthin Autofluorescence Using Flow Cytometry (Method A and B)

Daily plots of chlorophyll *a* fluorescence (excitation with blue laser at 488 nm with 690/50 optical filter) for assisting in cell counts using flow cytometry were also utilized on the day of harvest to retrieve single-cell chlorophyll fluorescence data. A secondary plot for detecting fucoxanthin was also created on this day to assess whether a different excitation/emission arrangement would be more reliable for detecting fucoxanthin than that used for detecting chlorophyll (488/690). Firstly, HPLC data was compared to data for all available flow cytometry channel arrangements, and the channel with highest correlation to fucoxanthin was chosen to be displayed. This was a yellow excitation laser at 561 nm with 710/50 nm optical filter. The mean single-cell fluorescence for both channels was used to compare to fucoxanthin as measured using HPLC. To explore whether chlorophyll *a* fluorescence measured using microplate reader could be sufficient to predict fucoxanthin content, daily chlorophyll *a* measurements from Section 2.3. were also compared to fucoxanthin measured using HPLC.

### 3.5. Nile Red Fluorescence Using Flow Cytometry (Method C)

Nile Red fluorescence using both microplate reader and flow cytometry was included to test the sensitivity of this dye in both formats. One milliliter of each diluted culture sample was dyed with 0.75 µg Nile Red for ~10 min, and fluorescence was measured using microplate reader Excitation/Emission wavelengths of 484/583 nm, while the cytometry equivalent was measured by retrieving the mean single cell fluorescence using blue excitation laser at 488 nm with 610/20 optical filter.

### 3.6. Ritchie (2008) Using Microplate Reader (Method D)

The equations for chlorophyll for Ritchie [18] were applied to extracts to test whether they could reliably predict fucoxanthin in ethanol:

Chl *a* = −0.9394 × A_632_ − 4.2774 × A_649_ + 13.3914 × A_665_

### 3.7. Wang et al. (2018) Using Microplate Reader (Method E)

A similar method to the equations above, yet for direct quantification of fucoxanthin in *P. tricornutum,* was also developed, albeit without the need for removal of cell debris after extraction [19]. The equation from the original source is as follows (Cfuc′ = Fucoxanthin concentration in mg L^−1^):

Cfuc′ = 6.39 × A_445_ − 5.18 × A_663_ + 0.312 × A_750_ − 5.27

### 3.8. Thrane et al. (2015) Using Microplate Reader (Method F)

The direct quantification method using visible light absorbance spectra developed by Thrane et al. [20], while requiring a more complex data analysis, was included to test the potential of ‘gauss-peak’ fitting originally established by Küpper, Spiller and Küpper [21]. The method is described thoroughly in Thrane et al. [20]. In short, individual pigment spectra are described by combinations of Gaussian peaks (termed the Gauss-peak spectra method) and are then modelled alongside background noise using non-negative least squares. The method utilizes R software to input coefficients for peak height, wavelength, and peak halfwidth to characterize pigments, and then compares this to sample absorbance spectra to estimate pigment content in mg L^−1^ using absorbance and pigment molar extinction coefficients. Thrane et al. [20] aimed to quantify multi-species pigment samples from lakes, whereas this work aims to adapt the method to a single species in a laboratory setting. The original source includes R scripts for readers to easily perform the method. To test if the method was further applicable to cultures without extraction, absorbance spectra obtained using diluted culture aliquots on a microplate reader were used instead of sample extract spectra.

### 3.9. HPLC for Pigment Detection

Freeze-dried pellet samples were weighed and re-suspended in a solvent ratio of between 2–3 mg of sample (DW) per mL of ethanol and sonicated with an ultrasonic homogenizer (Qsonica Q125) at 100% amplitude for 3 × 3-s pulses before being stored at −20 °C overnight once blanching of pellets was confirmed using a centrifuge. They were then filtered using 0.2 µm PTFE 13 mm syringe filters and stored in −80 °C until analysis. High Performance Liquid Chromatography (HPLC) was conducted using an Agilent Technologies 1290 Infinity, equipped with a binary pump with integrated vacuum degasser, thermostatted column compartment modules, Infinity 1290 auto-sampler, and PDA detector. Column separation was performed using a 4.6 mm × 150 mm Zorbax Eclipse XDB-C8 reverse-phase column (Agilent Technologies, Santa Clara, CA, USA) and guard column using a gradient of TBAA (tert-Butyl acetoacetate): Methanol mix (30:70) (solvent A) and Methanol (Solvent B) as follows: 0–22 min, from 5 to 95% B; 22–29 min, 95% B; 29–31 min, 5% B; and 31–40 min, column equilibration with 5% B. Column temperature was maintained at 55 °C. A complete pigment profile from 270 to 700 nm was recorded using PDA detector with 3.4 nm bandwidth. Example chromatograms can be found in supplementary files.

### 3.10. Additional Measurements

Media nitrate content was analyzed using an automated photometric analyzer (Gallery™ Discrete Analyzer, Thermo Fisher Scientific) by establishing standard calibration curves for nitrite and Total Oxidised Nitrogen (TON) from 0–20 mg L^−1^, after which samples from 10 × nitrate flasks were diluted to 1/20th of their original concentration to fit within this range. Spiked recoveries were performed using three samples with dissimilar concentrations, and these resulted in an average recovery of 100 ± 7.5%. 

### 3.11. Statistical Analysis

One-way ANOVA with a confidence level of 0.05 was performed followed by Tukey’s post-hoc to determine significance among samples, using GraphPad Prism version 9.0.2 for Windows (GraphPad Software, San Diego, CA, USA).

## 4. Conclusions

An increasing screening rate is a critical factor for high-throughput projects regarding microalgal libraries. In this aspect, the spectral deconvolution method from Thrane et al. [20] holds great potential because samples can be analyzed with temporal and multiple-condition evaluation of individuals whilst maintaining culture sterility. This method removes the cost of most expendables and reduces the time spent extracting samples, which also reduces the likelihood of variability introduced through solvent evaporation or pipetting. Most notably, once using modified input scripts, the spectral deconvolution method from Thrane et al. [20] provides researchers with accurate measurement of fucoxanthin in live cultures of *P. tricornutum*, which, to our knowledge, has never been achieved, and significantly improves the screening rate of novel strains for biotechnological purposes. While this work is limited to fucoxanthin in *P. tricornutum*, future projects should look at adapting spectral deconvolution to other microalgal species as well as other pigments of interest.

## Figures and Tables

**Table 1:** Practical considerations of tested high-throughput screens for fucoxanthin in *P. tricornutum.* Culture contact refers to removing vessel lids, pipetting, or transferring into measurement vessels.

Fucoxanthin Screening Method	Method Letter	Correlation to HPLC (R^2^)	No Dyeing Required	No Extraction Required	No Skilled Equipment Operators Required	No Culture Contact Required
Flow cytometry/FACS	A/B	0.949	✔	✔		
Nile Red	C	0.685		✔	✔	
Ritchie (2008) [18]	D	0.902	✔		✔	
Wang et al. (2018) [19]	E	0.859	✔		✔	
Thrane et al. (2015) [20]	F	0.849	✔	✔	✔	✔

## Data Availability

Not applicable.

## Supplementary Materials

The following are available online at https://www.mdpi.com/article/10.3390/md20010019/s1, Figure S1: Representative HPLC chromatograms of Phaeodactylum tricornutum extract from each treatment group. Treatment abbreviations are as follows: nitrate-free ASW media (-N), standard (1 × N) nitrate media, or media with 10× nitrate (10 × N) and either 10 (LL) or 200 (HL) µmol photons m^−2^ s^−1^. Peaks numbers are represented as follows: 1: Chlorophyll c; 2: Fucoxanthin; 3: Diadinoxanthin; 4: Chlorophyll a; 5: β-carotene.
