# Supplementary material for: Comparative Study Highlights the Potential of Spectral Deconvolution for Fucoxanthin Screening in Live Phaeodactylum tricornutum Cultures
Source: Mar Drugs. 2021 Dec 23;20(1):19. doi: 10.3390/md20010019 (PMC8780081; doi:10.3390/md20010019)
Supplement: Supplementary file 1 [file marinedrugs-20-00019-s001.zip › marinedrugs-1522621-supplementary.pdf]

Supplementary Materials:

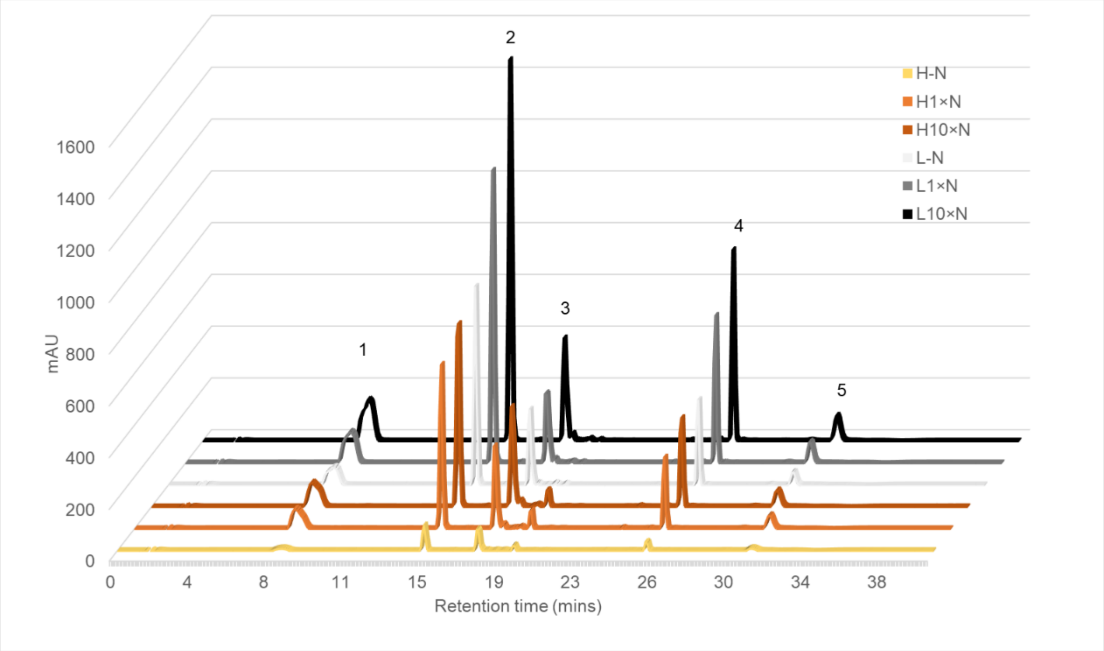

Figure S1. Representative HPLC chromatograms *of Phaeodactylum tricornutum* extract from each treatment group. Treatment abbreviations are as follows: nitrate-free ASW media (-N), standard (1xN) nitrate media, or media with 10× nitrate (10xN) and either 10 (LL) or 200 (HL) µmol photons m^{-2} s^{-1}. Peaks numbers are represented as follows: 1: Chlorophyll *c*; 2: Fucoxanthin; 3: Diadinoxanthin; 4: Chlorophyll *a*; 5: β-carotene.
